# Mortality in adult children of parents with alcohol use disorder: a nationwide register study

**DOI:** 10.1007/s10654-022-00883-4

**Published:** 2022-06-23

**Authors:** Jeanette Westman, Nitya Jayaram-Lindström, Kimberly Kane, Johan Franck, Mika Gissler

**Affiliations:** 1 Department of Neurobiology, Care Sciences and Society, Karolinska Institutet, Huddinge, Sweden; 2 Department of Health Care Sciences, Marie Cederschiöld University, Stockholm, Sweden; 3 Academic Primary Health Care Centre, Region Stockholm, Sweden; 4 Department of Clinical Neuroscience, Centre for Psychiatry Research, Karolinska Institutet, Stockholm, Sweden; 5 Department of Child Psychiatry, Turku University Hospital, Turku University, Turku, Finland; 6 Department of Knowledge Brokers, Finnish Institute for Health and Welfare, Helsinki, Finland

**Keywords:** Alcoholism, Substance use disorder, Addiction, Adult children of alcoholics

## Abstract

Research suggests that adult children of parents with harmful alcohol use are at increased risk for premature death. This national cohort study investigated mortality in adult children of parents with alcohol use disorder (AUD), adjusting for sociodemographic variables. The study used 1973 to 2018 data from Swedish national registers to compare mortality risk in children who had ≥ 1 parent with AUD (ICD-10 code F10 and its ICD-8 and ICD-9 equivalents) (n = 122,947) and those who did not (n = 2,298,532). A Cox regression model adjusted for year of birth, sex, parental education, and childhood loss of a parent was used. Before the age of 18 years, about 5% of children born in Sweden lived with ≥ 1 parent who had a clinical diagnosis of AUD. Overall mortality was higher in adult children of parents with AUD: hazard ratio (HR) 1.76, 95% confidence interval (CI) 1.71–1.82. Mortality remained elevated after adjustments for sociodemographic factors (HR 1.45, 95% CI 1.40–1.50). Children of parents with AUD had increased mortality from all investigated causes. The highest excess risk was for death from drug-related causes (excluding accidental poisonings) (HR 3.08, 95% CI 2.74–3.46). For most causes, mortality was higher if the mother had AUD than if the father had AUD. Patterns of mortality were similar in both sexes. This study provides evidence that parental AUD raises the risk of offspring mortality from preventable causes such as drug use, suicide (HR 2.16, 95% CI 1.98–2.36), accident (HR 2.00, 95% CI 1.87–2.13), and assault (HR 1.76, 95% CI 1.38–2.24).

**Supplementary Information:**

The online version contains supplementary material available at 10.1007/s10654-022-00883-4.

## Introduction

An estimated four million people in Western Europe and more than sixty million worldwide have alcohol use disorder (AUD) [1]. Millions of children, including approximately 4–10% of children in western countries [2–4], grow up with at least one parent with harmful alcohol use.

Not all children who grow up with a parent who has AUD are affected the same way or have negative outcomes [5–7]. However, adverse effects of having a parent with AUD are measurable from birth and range from somatic [2] and mental disorders [8] to increased mortality [2, 9]. Adult children of parents with AUD are at raised risk for substance use disorders [5, 10, 11], mental health problems [11, 12], violence [2], and suicide [13]. Previous studies also highlight that children who grow up with a parent who has AUD are at higher risk for outcomes that can negatively affect later socioeconomic position, such as lower levels of school performance [14], unemployment during their teen years and young adulthood [2], and teenage pregnancy [2]. Many of these outcomes are directly or indirectly associated with higher risks of mortality.

The few studies that have examined mortality risk in adult children of parents with AUD have found increased risk [2, 9]. A Danish prospective national birth cohort study found that parental alcohol abuse raised mortality risk in offspring up to the age of 27 and that maternal alcohol abuse had more impact than paternal alcohol abuse [2]. A survey study of Swedish men in compulsory military service found three times higher mortality risk in adult sons of fathers with AUD than in other survey respondents [9]. The same survey study found that mortality risk, including total mortality and mortality related to alcohol, violence, and suicide, was raised in conscripts who reported that their fathers drank often, including those whose fathers had no diagnosis of AUD [9].

Swedish national registers are highly complete and have been recording births [15], maternity [15], paternity [16], hospital diagnoses [17], and causes of death [16] for decades. Linking and analyzing these national population data can provide an overview of mortality in adult children of parents with AUD over more than four decades. The current study uses the data available in national registers to enhance existing information on mortality risk in adult children of parents with AUD.

## Aim

This national cohort study investigated mortality in adult children of parents with AUD, adjusting for year of birth, sex, level of parental education, and loss of a parent in childhood.

## Methods

### Study population and data sources

Data were obtained from national registers at the Swedish National Board of Health and Welfare and Statistics Sweden, the Swedish governmental statistics agency (Table 1). Individual-level data were linked via a pseudonymized version of the personal identification number assigned to each resident in Sweden.

**Table 1:** Sources of data in the study

Data	Source	Dates
Births	Medical Birth Register	1 Jan 1973–31 Dec 1995
Maternity	Medical Birth Register	1 Jan 1973–31 Dec 1995
Paternity	Total Population Register	1 Jan 1973–31 Dec 1995
Deaths (parents and offspring)	Total Population Register	1 Jan 1973–31 Dec 2018
Causes of death	Swedish National Cause of Death Register	1 Jan 1973–31 Dec 2018
Emigration	Total Population Register	1 Jan 1973–31 Dec 2018
Alcohol-related hospital diagnosis (parents and offspring)	Swedish National Patient Register	1 Jan 1973–31 Dec 2018
Level of education	Swedish census	1970, 1975, 1980, 1985, and 1990

We included all live-born children in Sweden born between January 1, 1973, and December 31, 1995 (n = 2,421,479), as well as information on their biological mothers (2,421,479, 100%) and fathers (n = 2,396,514, 99%). Those born between these dates were followed up until emigration, death, or the end of the study period on December 31, 2018, whichever came first.

Data on births and maternity came from the Swedish Medical Birth Register, established in 1973 and maintained by the Swedish National Board of Health and Welfare [15]. Data on paternity, deaths, and emigration for children and their parents came from the Swedish Total Population Register, established in 1968 and kept by Statistics Sweden [16].

Information on parents’ AUD before the birth of their child or when the child was 0 through 17 years (January 1, 1973, through December 31, 2013) was retrieved from the Swedish National Patient Register [17], kept by the National Board of Health and Welfare. This register started in the 1960s, when it covered mainly psychiatric care. It has included all inpatient hospital care in Sweden since 1987 [17]. Specialist psychiatric outpatient visits to both public and private service providers have been included since 2001.

Information on causes of death from 1973 through 2018 was obtained from the Cause of Death Register, maintained by the National Board of Health and Welfare. The medical death certificate is typically completed by the patient’s usual physician or the last physician to see the patient before death and is sent to the National Board of Health and Welfare within three weeks of the death. The main cause of death (ICD-8 from 1973 through 1986, ICD-9 from 1987 through 1996, and ICD-10 from 1997 through 2018) and contributing causes of death (up to 6 from 1973 through 1986, up to 12 from 1987 through 1996, and up to 48 from 1997 through 2018) are coded by the physician and checked by trained coders at the Cause of Death Register [18].

Information on the highest level of education achieved was obtained from census data, which were available for 1970, 1975, 1980, 1985, and 1990 [19]. We used the Swedish Educational Nomenclature (*Svensk utbildningsnomenklatur*) [20] to classify education by level. The classifications in the Swedish Educational Nomenclature have been harmonized with the 1997 International Standard Classification of Education.

### Variables

People with AUD are typically hospitalized during alcohol detoxification to prevent delirium tremens and seizures when heavy drinking is stopped abruptly. In this study, parental AUD was defined as a main or secondary hospital diagnosis of ICD-10 code F10 (mental and behavioral disorders due to use of alcohol) [21] or its ICD-8 [22, 23] and ICD-9 equivalents [23–25] (Additional file 1).

In children under 18 years, four categories of parental AUD were analyzed. 1) ≥ 1 parent had AUD, 2) at least the mother had AUD, 3) at least the father had AUD, and 4) both parents had AUD. The latter category was included because research shows that having two parents with AUD increases the risk for negative outcomes [26]. Children whose parents were diagnosed with AUD when the child was 18 years or older were included in the reference group. The reference group consisted of adult children whose parents did not receive a diagnosis of AUD before the child’s 18th birthday.

Causes of death (ICD-8, ICD-9, and ICD-10) were divided into any cause, medical causes (000–796/000–799/A00-R99), and external causes of injuries and poisoning (E840-E990/E840-E999/V01-Y89). External causes of injuries and poisoning were subdivided into suicide (E950-E959/E950-E959/X60-X84, Y87.0), assaults (E960-E969/E960-E969/X85-Y09, Y87.1), and accidents (E800-E949/E800-E990/V01-X59, Y85-Y86, Y10-Y15). We examined deaths related to the use of alcohol and drugs separately, dividing them into alcohol-related causes, drug-related causes, and drug-related causes excluding accidental poisonings (see Additional file 2 for a full list of ICD codes used in this separate analysis).

We also present a model adjusted for several sociodemographic variables, including year of birth (continuous, 1973–1995), sex (male or female), loss of ≥ 1 parent before the child turned 18 (yes or no), and highest level of parental education. The risk of losing a parent who has AUD is relatively high because addiction increases mortality risk [27–29]. Losing a parent in childhood or adolescence is a major adverse life event and has been associated with a higher likelihood of unemployment in adult men [30], as well as short, medium, and long-term negative mental health outcomes, such as depression and suicide [31]. Highest level of parental education (mother’s or father’s, whichever was highest) (1970–1990) was divided into basic (up to 9 years), secondary (10–12 years), tertiary (13 or more years), and missing.

In an additional analysis, we investigated the associations between parental AUD and mortality separately for sons and daughters. This analysis was undertaken because some studies have found that the sex of the parent with AUD and their offspring is differentially associated with psychiatric morbidity and negative social and behavioral outcomes from childhood through early adulthood [2, 12, 32, 33], whereas other studies have not observed such sex-based associations [10, 34–36].

### Statistical methods

Differences between children whose parents did and did not have AUD were tested with *t*- and chi-square tests. Cox regression models with 95% confidence intervals were used to compare the mortality in children whose parents did and did not have AUD. Follow-up started at birth and ended at death, migration from Sweden, or December 31, 2018, whichever came first.

Mortality rates were calculated per 100,000 follow-up years. After confirming that the assumptions for Cox regression were fulfilled, both crude and background-adjusted hazard ratios (HRs) with 95% confidence intervals (CIs) were calculated. HRs were adjusted for year of birth, sex, highest level of parental education, and loss of ≥ 1 parent before the child reached the age of 18 years. A sensitivity analysis that included only the first births of each mother-father pair (firstborns) was performed to test whether the inclusion of siblings (dependent observations) affected the results of the Cox regression.

The data analysis for this paper was generated using SAS/STAT software, Version 9.4 of the SAS System for Windows 7 (SAS Institute Inc., Cary, NC, USA).

## Results

A total of 122,947 people in the study population (5.1%) had ≥ 1 parent with AUD (Table 2). The number who had a father with AUD (87,101; 3.6%) was higher than the number who had a mother with the disorder (43,038; 1.8%). Because of the relatively small number whose mother and father both had AUD (n = 7192, 0.30%), this subgroup had few deaths from some of the causes investigated in this study. HRs of death for adult children whose parents both had AUD are therefore not presented (Tables 5, 6, 7, 8 and Additional file 3).

**Table 2:** Characteristics of the study population^a^

	All	Neither parent had AUD	≥ 1 parent had AUD	Both parents had AUD	Mother had AUD	Father had AUD	Son of ≥ 1 parent with AUD	Daughter of ≥ 1 parent with AUD
Number	2,421,479	2,298,532	122,947	7192	43,038	87,101	63,024	59,923
%	100%	94.9%	5.1%	0.30%	1.8%	3.6%	5.1%	5.1%
*Sex*								
Male	1,244,135	1,181,111	63,024	3616	22,113	44,527	63,024	N/A
	51.4%	51.4%	51.3%	50.3%	51.4%	51.1%	51.3%	N/A
Female	1,177,344	1,117,421	59,923	3576	20,925	44,527	N/A	59,923
	48.6%	48.6%	48.7%	49.7%	48.6%	48.9%	N/A	48.7%
Death of one or both parents before offspring turned 18 years	82,122	66,387	15,735	1788	5050	12,473	8027	7708
	3.4%	2.9%	12.8%	24.9%	11.7%	14.3%	12.7%	12.9%
*Highest level of parental education*^b^								
Basic	520,057	481,569	38,488	2613	12,763	28,338	19,611	18,877
	21.5%	21.0%	31.3%	36.3%	29.7%	32.5%	31.1%	31.5%
Secondary	804,441	761,292	43,149	2209	15,193	30,165	22,148	21,001
	33.2%	33.1%	35.1%	30.7%	35.3%	34.6%	35.1%	35.0%
Tertiary	893,791	872,066	21,725	643	8507	13,861	11,157	10,568
	36.9%	37.9%	17.7%	8.9%	19.8%	15.9%	17.7%	17.6%
Missing	203,190	183,605	19,585	1727	6575	14,737	10,108	9477
	8.4%	8.0%	15.9%	24.0%	15.3%	16.9%	16.0%	15.8%

N/A: not applicable

^a^Live-born children in Sweden born between January 1, 1973, and December 31, 1995

^b^Mother’s or father’s, whichever was highest; basic: up to 9 years, secondary: 10 to 12 years, tertiary: 13 or more years

In 35.2% of cases (43,307 of 122,947), ≥ 1 parent received the diagnosis during or before pregnancy. More of these early diagnoses were received by mothers (37.5%, 16,141 of 43,038 diagnoses) than fathers (33.3%, 29,030 of 87,101) (*P* < 0.001).

The sex distribution was equal in children of parents with and without AUD (*P* = 0.395) (Table 2). More children with ≥ 1 parent who had AUD lost a parent before they turned 18 years (*P* < 0.001). Additionally, a higher proportion had parents with only a basic education (≤ 9 years) (*P* < 0.001).

During the follow-up period, there were 43,153 deaths in the study population: 3731 in those with ≥ 1 parent with AUD and 39,422 in those whose parents did not have AUD (Table 3). Mortality rates followed a consistent pattern across all investigated causes of death (Table 4). The highest rates were observed in adult children whose parents both had AUD, followed by those whose mother had AUD, those whose father had AUD, and those who did not have a parent with AUD. Mortality rates for deaths from any cause ranged from 54 per 100,000 person-years in adult children whose parents did not have AUD to 136 per 100,000 person-years in adult children whose parents both had AUD.

**Table 3:** Number of deaths in the study population^a^ by cause and parental alcohol use disorder

	All	Neither parent had AUD	≥ 1 parent had AUD	Both parents had AUD	Mother had AUD	Father had AUD
Number	2,421,479	2,298,532	122,947	7192	43,038	87,101
Cause of death						
Any cause	43,153	39,422	3731	318	1515	2534
Medical causes	28,047	26,182	1865	133	766	1232
External causes of injuries and poisoning	15,466	13,599	1867	186	750	1303
Suicide	5113	4497	616	63	262	417
Assault	619	538	81	8	33	56
Accident	9734	8564	1170	115	455	830
Alcohol-related causes	197	175	22	5	9	18
Drug-related causes	4411	3645	766	86	294	558
Drug-related causes excluding accidental poisonings^b^	2144	1764	380	44	153	271

^a^Live-born children in Sweden born between January 1, 1973, and December 31, 1995

^b^Accidental poisonings: ICD-8: 304.00–304.30; ICD-9: E8500, E851, E8530; ICD-10: X41-X44

**Table 4:** Mortality per 100,000 person-years in the study population^a^ by cause and parental alcohol use disorder

	All	Neither parent had AUD	≥ 1 parent had AUD	Both parents had AUD	Mother had AUD	Father had AUD
Number	2,421,479	2,298,532	122,947	7192	43,038	87,101
Cause of death						
Any cause	56	54	95	136	112	90
Medical causes	36	36	47	57	56	44
External causes of injuries and poisoning	20	18	47	80	55	46
Suicide	7	6	16	27	19	15
Assault	0.8	0.7	2.1	3.4	2.4	2.0
Accident	13	12	30	49	34	29
Alcohol-related causes	0.3	0.2	0.6	2.1	0.7	0.6
Drug-related causes	6	5	19	37	22	20
Drug-related causes excluding accidental poisonings^b^	3	2	10	19	11	10

^a^Live-born children in Sweden born between January 1, 1973, and December 31, 1995

^b^Accidental poisonings: ICD-8: 304.00–304.30; ICD-9: E8500, E851, E8530; ICD-10: X41-X44

Crude HRs of mortality from all investigated causes were higher in those with ≥ 1 parent who had AUD than those whose parents did not have the disorder (Table 5). For death from any cause, the HR in children with ≥ 1 parent with AUD was 1.76 (95% CI 1.71–1.82). Across all causes of death examined in the study, risk was highest when both parents had AUD (data not shown) and higher for maternal AUD (HR of death from any cause: 2.04, 95% CI 1.94–2.15) than paternal AUD (HR of death from any cause: 1.65, 95% CI 1.59–1.72).

**Table 5:** Crude hazard ratios and 95% confidence intervals of death in the study population^a^ (n = 2,421,479) by cause and parental alcohol use disorder

	Neither parent had AUD	≥ 1 parent had AUD	Mother had AUD	Father had AUD
Number	2,298,532	122,947^b^	43,038	87,101
Cause of death				
Any cause	1.0	1.76 (1.71–1.82)	2.04 (1.94–2.15)	1.65 (1.59–1.72)
Medical causes	1.0	1.34 (1.28–1.40)	1.58 (1.47–1.70)	1.23 (1.16–1.30)
External causes of injuries and poisoning	1.0	2.58 (2.46–2.71)	2.90 (2.70–3.12)	2.44 (2.31–2.59)
Suicide	1.0	2.59 (2.38–2.81)	3.11 (2.74–3.52)	2.36 (2.14–2.61)
Assault	1.0	2.82 (2.23–3.56)	3.17 (2.23–4.50)	2.64 (2.01–3.48)
Accident	1.0	2.56 (2.41–2.72)	2.78 (2.53–3.05)	2.47 (2.30–2.65)
Alcohol-related causes	1.0	2.42 (1.55–3.77)	2.84 (1.45–5.54)	2.72 (1.67–4.41)
Drug-related causes	1.0	3.99 (3.69–4.31)	4.16 (3.69–4.68)	3.86 (3.53–4.22)
Drug-related causes excluding accidental poisonings^c^	1.0	4.09 (3.66–4.57)	4.47 (3.79–5.27)	3.86 (3.39–4.38)

^a^Live-born children in Sweden born between January 1, 1973, and December 31, 1995

^b^For 7192 children, both parents had AUD

^c^Accidental poisonings: ICD-8: 304.00–304.30; ICD-9: E8500, E851, E8530; ICD-10: X41-X44

For most outcomes, excess mortality decreased but remained statistically significant after adjustment for sociodemographic variables, and maternal AUD remained associated with higher excess risk than paternal AUD (Table 6). However, after adjustment, risk of death from medical causes was significant only for maternal AUD (maternal AUD: HR 1.37, 95% CI 1.27–1.47; paternal AUD: HR 1.01, 95% CI 0.96–1.07). Patterns of risk in the separate analysis of firstborns (Additional file 3) were consistent with those in the analysis of adjusted HRs in the whole study population (Table 6).

**Table 6:** Adjusted^a^ hazard ratios and 95% confidence intervals of death in the study population^b^ (n = 2,421,479) by cause and parental alcohol use disorder

	Neither parent had AUD	≥ 1 parent had AUD	Mother had AUD	Father had AUD
Number	2,298,532	122,947^c^	43,038	87,101
Cause of death				
Any cause	1.0	1.45 (1.40–1.50)	1.71 (1.63–1.80)	1.32 (1.27–1.38)
Medical causes	1.0	1.13 (1.08–1.18)	1.37 (1.27–1.47)	1.01 (0.96–1.07)
External causes of injuries and poisoning	1.0	2.04 (1.94–2.14)	2.32 (2.16–2.50)	1.87 (1.76–1.98)
Suicide	1.0	2.16 (1.98–2.36)	2.61 (2.30–2.96)	1.91 (1.72–2.12)
Assault	1.0	1.76 (1.38–2.24)	2.08 (1.46–2.96)	1.56 (1.17–2.07)
Accident	1.0	2.00 (1.87–2.13)	2.20 (2.00–2.42)	1.87 (1.73–2.01)
Alcohol-related causes	1.0	1.84 (1.17–2.92)	2.21 (1.13–4.35)	2.02 (1.23–3.34)
Drug-related causes	1.0	3.02 (2.78–3.28)	3.13 (2.78–3.53)	2.80 (2.55–3.08)
Drug-related causes excluding accidental poisonings^d^	1.0	3.08 (2.74–3.46)	3.36 (2.84–3.97)	2.78 (2.43–3.17)

^a^Adjusted for year of birth, sex, highest level of parental education, and loss of a parent in childhood

^b^Live-born children in Sweden born between January 1, 1973, and December 31, 1995

^c^For 7192 children, both parents had AUD

^d^Accidental poisonings: ICD-8: 304.00–304.30; ICD-9: E8500, E851, E8530; ICD-10: X41-X44

In a supplementary analysis of medical causes of death (data not shown), we found that among children who had experienced parental AUD, mortality risk due to mental and behavioral disorders was raised (ICD-8 codes 290–315, ICD-9 codes 290–319, ICD-10 codes F00-F99; adjusted HR 4.05; 95% CI 3.04–5.41; n = 305). The same was true of mortality risk due to symptoms, signs, and abnormal clinical and laboratory findings (ICD-8 codes 780–796, ICD-9 codes 780–799, ICD-10 codes R00-R99; adjusted HR 1.88; 95% CI 1.65–2.14; n = 2628) and endocrine, nutritional, and metabolic diseases (ICD-8 and ICD-9 codes 240–279, ICD-10 codes E00-E90; HR 1.55; 95% CI 1.25–2.00; n = 949). Children with ≥ 1 parent with AUD had a substantially increased risk of death from sudden infant death syndrome (no ICD-8 code for this disorder, ICD-9 code 7980, ICD-10 code R95), which was highest if the mother had AUD (adjusted HR 3.58, 95% CI 2.71–4.72, n = 857).

Those who had ≥ 1 parent with AUD had twice the risk of dying from external causes of injuries and poisoning, a category that includes suicide, assault, and accident (HR 2.04, 95% CI 1.94–2.14) (Table 6). Having a mother with AUD conferred greater excess risk (HR 2.32, 95% CI 2.16–2.50) than having a father with AUD (HR 1.87, 95% CI 1.76–1.98). Excess mortality due to suicide was approximately double that of those whose parents did not have AUD (HR 2.16, 95% CI 1.98–2.36), and the same was true of excess mortality due to accidents (HR 2.00, 95% CI 1.87–2.13). Those with ≥ 1 parent who had AUD also had an elevated risk of death due to assault (HR 1.76, 95% CI 1.38–2.24).

Adult children with ≥ 1 parent with AUD had triple the risk of dying from drug-related causes than those whose parents did not have the disorder (drug-related causes: HR 3.02, 95% CI 2.78–3.28; drug-related causes excluding accidental poisonings: HR 3.08, 95% CI 2.74–3.46) (Table 6). In this relatively young cohort, there were fewer deaths from alcohol-related than drug-related causes. Nevertheless, those with ≥ 1 parent with AUD had a higher risk of mortality from alcohol-related causes (HR 1.84, 95% CI 1.17–2.92) than those whose parents did not have AUD. In the analysis of firstborns only (Additional file 3), the adjusted HRs of death from alcohol-related causes were no longer significantly higher in any group of adult children of parents with AUD than in adult children whose parents did not have the disorder.

Mortality risk by the different causes of death was similar for sons (Table 7) and daughters (Table 8) of parents with AUD. The exception was risk of death from any cause, which was significantly more elevated in sons whose fathers had AUD (HR 1.43, 95% CI 1.36–1.50) than in daughters whose fathers had AUD (HR 1.14, 95% CI 1.06–1.23).

**Table 7:** Adjusted^a^ hazard ratios and 95% confidence intervals of mortality by causes of death in sons in the study population^b^ who had ≥ 1 parent with alcohol use disorder

	Neither parent had AUD	≥ 1 parent had AUD	Mother had AUD	Father had AUD
Number in the study population	2,298,532	122,947^c^	43,038	87,101
Sons	1,181,111	63,024	22,113	44,527
Cause of death				
Any cause	1.0	1.52 (1.46–1.59)	1.71 (1.60–1.82)	1.43 (1.36–1.50)
Medical causes	1.0	1.16 (1.09–1.23)	1.32 (1.20–1.45)	1.08 (1.00–1.16)
External causes of injuries and poisoning	1.0	2.04 (1.92–2.16)	2.24 (2.05–2.44)	1.91 (1.78–2.04)
Suicide	1.0	2.08 (1.87–2.31)	2.40 (2.05–2.80)	1.89 (1.67–2.14)
Assault	1.0	1.83 (1.36–2.45)	2.22 (1.45–3.39)	1.67 (1.19–2.35)
Accident	1.0	2.03 (1.89–2.19)	2.17 (1.94–2.42)	1.93 (1.78–2.10)
Alcohol-related causes	1.0	1.65 (0.91–2.99)	1.11 (0.35–3.51)	2.08 (1.12–3.85)
Drug-related causes	1.0	3.04 (2.77–3.34)	2.88 (2.50–3.33)	2.93 (2.63–3.25)
Drug-related causes excluding accidental poisonings^d^	1.0	3.16 (2.74–3.65)	2.97 (2.39–3.69)	3.02 (2.57–3.54)

^a^Adjusted for year of birth, sex, highest level of parental education, and loss of a parent in childhood

^b^Live-born children in Sweden born between January 1, 1973, and December 31, 1995 (n = 2,421,479)

^c^For 7192 children (3616 sons), both parents had AUD

^d^Accidental poisonings: ICD-8: 304.00–304.30; ICD-9: E8500, E851, E8530; ICD-10: X41-X44

**Table 8:** Adjusted^a^ hazard ratios and 95% confidence intervals of mortality by causes of death in daughters in the study population^b^ who had ≥ 1 parent with alcohol use disorder

	Neither parent had AUD	≥ 1 parent had AUD	Mother had AUD	Father had AUD
Number in the study population	2,298,532	122,947^c^	43,038	87,101
Daughters	1,117,421	59,923	20,925	42,574
Cause of death				
Any cause	1.0	1.33 (1.25–1.41)	1.73 (1.59–1.88)	1.14 (1.06–1.23)
Medical causes	1.0	1.09 (1.01–1.18)	1.43 (1.28–1.59)	0.93 (0.84–1.02)
External causes of injuries and poisoning	1.0	2.04 (1.85–2.25)	2.57 (2.23–2.95)	1.76 (1.57–1.98)
Suicide	1.0	2.36 (2.02–2.76)	3.10 (2.50–3.84)	1.97 (1.63–2.37)
Assault	1.0	1.62 (1.06–2.48)	1.81 (0.95–3.44)	1.35 (0.81–2.25)
Accident	1.0	1.88 (1.65–2.15)	2.31 (1.91–2.80)	1.67 (1.43–1.95)
Alcohol-related causes	1.0	2.22 (1.08–4.58)	4.41 (1.88–10.32)	1.93 (0.82–4.57)
Drug-related causes	1.0	2.96 (2.51–3.50)	3.92 (3.14–4.89)	2.44 (2.01–2.97)
Drug-related causes excluding accidental poisonings^d^	1.0	2.93 (2.40–3.58)	4.10 (3.16–5.33)	2.35 (1.85–2.98)

^a^Adjusted for year of birth, sex, highest level of parental education, and loss of a parent in childhood

^b^Live-born children in Sweden born between January 1, 1973, and December 31, 1995 (n = 2,421,479)

^c^For 7192 children (3576 daughters), both parents had AUD

^d^Accidental poisonings: ICD-8: 304.00–304.30; ICD-9: E8500, E851, E8530; ICD-10: X41-X44

## Discussion

### Main findings

Prior to age 18, one in 20 children in Sweden lived with ≥ 1 parent whose addiction to alcohol was severe enough to lead to a clinical diagnosis of AUD. Mortality risk was significantly higher in those whose parents had a registered clinical diagnosis of AUD than in those whose parents did not have such a diagnosis, even after adjustment for year of birth, sex, highest level of parental education, and loss of a parent in childhood. The data showed a pattern of higher risk when mothers had AUD than when fathers had the disorder. Those with ≥ 1 parent with AUD had approximately twice the risk of dying from external causes of injuries and poisoning than those whose parents did not have AUD. Excess mortality risk from external causes of injuries and poisonings was highest from suicide, followed by accidents and assaults. There were more deaths due to drugs than alcohol, possibly because of the relatively young age of the study population. Mortality from drug-related causes in people with ≥ 1 parent who had AUD was three times as high as in those whose parents did not have the disorder. Patterns of mortality were similar in offspring of both sexes, although sons of fathers with AUD had significantly greater excess risk of dying from any cause than daughters of fathers with AUD.

### Strengths and limitations

The national registers used in the current study have several strengths. Coverage in the Medical Birth Register is almost complete (1% to 4% missing data per year), and the quality of the register is high [37]. The Total Population Register has complete coverage of people registered as resident in Sweden [38]. The proportion of the Swedish population living in counties that reported psychiatric hospital discharges to the Swedish Inpatient Register grew over time from 86% in 1973 to 94% in 1985 and has been 98% or above since 1986 [39]. Linkage between different data sources was facilitated by the personal identification number assigned to every resident in Sweden and used in all Swedish nationwide health and sociodemographic registers.

As in all cohort studies, any conclusions about causality must be drawn with care and viewed as hypotheses. Moreover, the national registers used in the present study have limitations. For instance, they do not cover lifestyle factors relevant to mortality, such as smoking, diet, and physical activity, or variables potentially related to resilience, such as close sibling relationships or membership in support groups. Additionally, in this study, we did not have the opportunity to link the register data with information on the children’s residence, that is, to include information on which parent(s) the children lived with. A further limitation is the relatively young age of the study population at the end of follow-up. Because the oldest participants had not yet reached their 50s by the end of the study period, the data likely underestimate the effects of parental AUD on causes of death that are common later in life, such as cardiovascular diseases and alcohol-related causes (e.g., liver cirrhosis). The relatively young age of the study population also meant that it was not meaningful to calculate life expectancies; the differences between people with or without a parent who had AUD were small because of low mortality in people younger than 50. For example, for those born in 1973, the difference in life expectancy was 0.3 years for men and 0.1 years for women. Additionally, it should be noted that register studies with multiple outcomes are vulnerable to problems related to multiple testing. As discussed in Bender and Lange 2001 [40], adjustment for multiple comparison is not strictly necessary in studies of the current type, but it is important to note that the results are exploratory.

The National Patient Register only includes AUD diagnoses from hospital inpatient and (for later dates) specialized psychiatric outpatient care. Thus, the actual population of children exposed to parental AUD or problematic alcohol use is likely larger than captured in this study, as the data did not include parents who received treatment in primary health care, social care, or support groups such as Alcoholics Anonymous [41]. Our results should therefore be interpreted as reflecting mortality in the offspring of those most severely affected by AUD; we do not know how the inclusion of less severe but still problematic parental alcohol use would have affected the results. Additionally, many people have harmful alcohol use for extended periods of time before they receive treatment for AUD [42–44]. It is therefore possible that we underestimated parental AUD for children born in the latter part of the study period, as some parents with AUD may not yet have developed symptoms severe enough to receive a hospital diagnosis.

### Comparison with previous studies

#### Proportion of parents with alcohol use disorder in previous studies

The results showed that in absolute numbers, many children in Sweden have a parent with severe AUD. The proportion of children with ≥ 1 parent who had AUD in this study, 5.1%, was higher than that observed in other register-based estimates from the Nordic countries. For instance, a 2013 Swedish report based on national register data found that 2.5% of children born between 1987 and 1989 had at least one parent who had been hospitalized for alcohol abuse [45]. It is possible that birth cohort differences, differences in the ICD codes used in the two studies, or both, explain the divergent findings. The proportion of children in the current study who have experienced parental AUD is relatively comparable to that observed in a Danish register study that investigated a 1966 birth cohort and their parents [2]. That study, which used proxies for alcohol abuse similar but not identical to those in the current study, found that 4.5% of the children had experienced parental alcohol abuse. Earlier survey-based estimates have ranged from 3.7% in Sweden [3] to 4% in Norway [46], 6% in the United Kingdom [47], and 10% in the United States [4]. Differences in study designs, periods, and populations, as well as differences in the prevalence of alcohol use and abuse around the world [1] may play a role in these varying estimates.

#### Mortality risk in people with at least one parent with alcohol use disorder

We found a higher risk of mortality in people whose parent(s) had AUD even after controlling for several risk factors, such as loss of a parent before the child turned 18 and the family’s socioeconomic position. This is in keeping with the findings of previous studies [2, 9]. People with ≥ 1 parent with AUD often face hindrances to a healthy future. For example, there are genetic correlations between alcohol dependence and schizophrenia, ADHD, and depression, as well as cigarette and cannabis use [48]. Other disorders associated with alcohol abuse, such as anxiety and “antisocial or undercontrolled behavior” [5] have a heritable component [49, 50] and are themselves associated with increased mortality [51, 52]. Thus, inheritance and psychiatric morbidity are important potential explanations of increased mortality in adult children of parents with AUD.

Social disadvantages that can both contribute to [53] and be caused by [54] AUD, such as unemployment or homelessness, could have played a role in our findings. We controlled for parental education and death of a parent during childhood. However, we could not control for other social and emotional adverse experiences that could worsen the consequences of growing up with a parent with AUD [6], such as abuse, neglect, illness, or encounters with law enforcement.

#### Sex of the parent with alcohol use disorder

AUD is more prevalent in men than women [55]. In keeping with this, in the current study, as in previous studies [2, 45], it was more common to have a father than a mother with AUD. However, adjusted risk of mortality was generally higher when the mother had AUD than when the father had the disorder. This result is consistent with the results of other register studies. For instance, a Danish study found that maternal rather than paternal alcohol abuse was more frequently associated with all the negative long-term consequences they tracked in offspring, including mortality, attempted suicide, and hospitalization due to violence [2]. The same pattern emerged from a Swedish study of risk for externalizing psychopathology (defined as AUD, criminal behavior, drug abuse, and ADHD) in children of parents with AUD [12]. In that study, stratified analyses showed that contact with the affected parent (most often the mother) played a prominent explanatory role. A similar explanation may underlie our findings. Even in Sweden, where gender equality is a goal of government policy, mothers are often still children’s primary caregivers [56]. Approximately two-thirds of mothers but only one-third of fathers with a hospital diagnosis of substance abuse (including but not limited to alcohol abuse) live with their children [45]. AUD can also disrupt family structures, and it is more common for children of parents with AUD than children of parents without AUD to grow up with only one or neither of their parents [57].

#### Excess mortality by sex of offspring

We found one difference in mortality risk by the sex of the offspring of parents with AUD: when fathers had AUD, risk of death from any cause was significantly more elevated in sons than in daughters. Previous studies differ in their findings about whether the sex of the child who experienced parental AUD is related to negative social and behavioral outcomes from childhood through early adulthood [2, 12, 32, 33] or is not related to such outcomes [10, 34–36]. Like the current study, studies that observe such a relationship have found that both the sex of the parent and the sex of the child are relevant [2, 12, 32, 33]. For instance, a nationwide Swedish study that used data from a broader range of sources than the current study found that when fathers had AUD, the risk of AUD and of criminal behavior was raised more in sons than daughters [12]. Those researchers also found that when mothers had AUD, the risk of drug abuse was raised more in daughters than sons.

#### Risk of dying from external causes

Excess mortality risk from external causes was highest from suicide, followed by accidents and assaults. This is consistent with the findings of a Swedish study of men in obligatory military service, which found a higher risk for alcohol-related mortality, suicide, and violent death in men who reported that their fathers drank occasionally or often [9]. In that study, the raised risk of death due to suicide and violence was largely explained by problematic alcohol use, smoking, mental disorders, emotional instability, and encounters with law enforcement and civil authorities [9]. A register study from Denmark that found increased mortality in 15- to 27-year old offspring of parents who abused alcohol did not investigate causes of death but found an increased risk of hospitalization due to violence, as well as increases in attempted suicide [2].

#### Risk of drug- and alcohol-related mortality

We found a three-fold increased risk of drug-related mortality and an excess risk of alcohol-related mortality in children with ≥ 1 parent with AUD. The raised risk of alcohol-related mortality is consistent with the findings of the survey-based study of young men conscripted to the military in Sweden, which found a raised risk of alcohol-related mortality in sons who reported that their fathers used alcohol [9]. However, in the supplementary analyses of firstborns, there was no increased risk of mortality from alcohol-related causes in children with ≥ 1 parent with AUD. The relatively young age of our study population may have contributed to the relatively low number of alcohol-related deaths. With increasing age, more people in the study population may develop the long-term somatic consequences of harmful alcohol use, such as cardiovascular disease [58], diabetes [58], cancers [58], liver and pancreatic diseases [58], or dementia [59, 60].

## Conclusions

This nationwide register study shows that parental AUD raises the risk of offspring mortality from preventable causes such as drug use, suicide, accident, and assault. These findings highlight the potential adversity of growing up in a family affected by AUD.

## Supplementary Information

Below is the link to the electronic supplementary material.

Supplementary file1 (DOCX 25 KB)

Supplementary file2 (DOCX 28 KB)

Supplementary file3 (DOCX 23 KB)

## Data Availability

Access to the national register data used in this study can be requested from the Swedish National Board of Health and Welfare and Statistics Sweden.

## References

[1] Peacock A, Leung J, Larney S, Colledge S, Hickman M, Rehm J (2018). Global statistics on alcohol, tobacco and illicit drug use: 2017 status report. Addiction.

[2] Christoffersen MN, Soothill K (2003). The long-term consequences of parental alcohol abuse: a cohort study of children in Denmark. J Subst Abuse Treat.

[3] Raninen J, Elgán TH, Sundin E, Ramstedt M (2016). Prevalence of children whose parents have a substance use disorder: findings from a Swedish general population survey. Scand J Public Health.

[4] Substance Abuse and Mental Health Services Administration (SAMHSA). Data Spotlight: More than 7 Million Children Live with a Parent with Alcohol Problems. In: Center for Behavioral Health Statistics and Quality USDoHaHS, editor.2012.

[5] Harter SL (2000). Psychosocial adjustment of adult children of alcoholics: a review of the recent empirical literature. Clin Psychol Rev.

[6] Hall CW, Webster RE (2007). Risk factors among adult children of alcoholics. Int J Behav Consult Ther.

[7] Miller JB. Discovering Happiness: A New Approach to Understanding Adult Children of Alcoholics [Psy.D.]. Ann Arbor: The Chicago School of Professional Psychology; 2016.

[8] Raitasalo K, Holmila M, Jääskeläinen M, Santalahti P (2019). The effect of the severity of parental alcohol abuse on mental and behavioural disorders in children. Eur Child Adolesc Psychiatry.

[9] Landberg J, Danielsson AK, Falkstedt D, Hemmingsson T (2018). Fathers' alcohol consumption and long-term risk for mortality in offspring. Alcohol Alcohol.

[10] Mellentin AI, Brink M, Andersen L, Erlangsen A, Stenager E, Bjerregaard LB (2016). The risk of offspring developing substance use disorders when exposed to one versus two parent(s) with alcohol use disorder: a nationwide, register-based cohort study. J Psychiatr Res.

[11] Holst C, Tolstrup JS, Sørensen HJ, Pisinger VSC, Becker U (2019). Parental alcohol use disorder with and without other mental disorders and offspring alcohol use disorder. Acta Psychiatr Scand.

[12] Long EC, Lönn SL, Sundquist J, Sundquist K, Kendler KS (2018). The role of parent and offspring sex on risk for externalizing psychopathology in offspring with parental alcohol use disorder: a national Swedish study. Soc Psychiatry Psychiatr Epidemiol.

[13] Landberg J, Danielsson AK, Hemmingsson T (2019). Fathers' alcohol use and suicidal behaviour in offspring during youth and young adulthood. Acta Psychiatr Scand.

[14] Berg L, Bäck K, Vinnerljung B, Hjern A (2016). Parental alcohol-related disorders and school performance in 16-year-olds-a Swedish national cohort study. Addiction.

[15] Swedish National Board of Health and Welfare. The Swedish Medical Birth Register. 2019. https://www.socialstyrelsen.se/en/statistics-and-data/registers/register-information/the-swedish-medical-birth-register/. Accessed 2 July 2020.

[16] Sweden S. Microdata for the Total Population Register. Statistics Sweden. n.d. https://www.scb.se/vara-tjanster/bestalla-mikrodata/vilka-mikrodata-finns/individregister/registret-over-totalbefolkningen-rtb/. Accessed 15 December 2020.

[17] Swedish National Board of Health and Welfare. The National Patient Register. 2019. https://www.socialstyrelsen.se/en/statistics-and-data/registers/register-information/the-national-patient-register/. Accessed 2 July 2020.

[18] Brooke HL, Talbäck M, Hörnblad J, Johansson LA, Ludvigsson JF, Druid H (2017). The Swedish cause of death register. Eur J Epidemiol.

[19] Statistics Sweden. Description of archived files. 2008. https://web.archive.org/web/20100223100344/http://www.scb.se/Pages/List____250827.aspx. Accessed 10 August 2020.

[20] Öberg S, Olsson AK. Swedish Educational Nomenclature. Statistics Sweden; 2000.

[21] World Health Organization. International Statistical Classification of Diseases and Related Health Problems 10th Revision (Browser). 2019. https://icd.who.int/browse10/2019/en. Accessed May 17 2021.

[22] Swedish National Board of Health and Welfare. ICD-8 Classification of diseases etc. 1968 (1969–1986) codes and code text in Excel format. In: Historical classifications. Swedish National Board of Health and Welfare. 2019. https://www.socialstyrelsen.se/utveckla-verksamhet/e-halsa/klassificering-och-koder/icd-10/historiska-klassifikationer/. Accessed May 23, 2021.

[23] van Drimmelen J (1994). The ICD-10 classification of mental and behavioural disorders: conversion tables between ICD-8, ICD-9 and ICD-10.

[24] Swedish National Board of Health and Welfare. ICD-9 Classification of diseases 1987 (KS87) (1987–1996) codes and code text in Excel format. In: Historic classifications (ICD). Swedish National Board of Health and Welfare. 2019. https://www.socialstyrelsen.se/utveckla-verksamhet/e-halsa/klassificering-och-koder/icd-10/historiska-klassifikationer/. Accessed 10 May 2021.

[25] Swedish National Board of Health and Welfare. Classification of Diseases 1987: Systematic List - Swedish version of the International Classification of Disease, Ninth Revision (ICD-9). Stockholm Liber; 1986.

[26] Kosty DB, Farmer RF, Seeley JR, Merikangas KR, Klein DN, Gau JM (2020). The number of biological parents with alcohol use disorder histories and risk to offspring through age 30. Addict Behav.

[27] Kendler KS, Ohlsson H, Sundquist J, Sundquist K (2016). Alcohol use disorder and mortality across the lifespan: a longitudinal cohort and co-relative analysis. JAMA Psychiat.

[28] Laramée P, Leonard S, Buchanan-Hughes A, Warnakula S, Daeppen JB, Rehm J (2015). Risk of all-cause mortality in alcohol-dependent individuals: a systematic literature review and meta-analysis. EBioMedicine.

[29] Roerecke M, Rehm J (2013). Alcohol use disorders and mortality: a systematic review and meta-analysis. Addiction.

[30] Parsons S. Long-term impact of childhood bereavement Preliminary analysis of the 1970 British Cohort Study In: Education Df, editor. United Kingdom: Childhood Wellbeing Research Centre; 2011.

[31] Feigelman W, Rosen Z, Joiner T, Silva C, Mueller AS (2017). Examining longer-term effects of parental death in adolescents and young adults: Evidence from the national longitudinal survey of adolescent to adult health. Death Stud.

[32] Marino EN, Fromme K (2015). Alcohol-induced blackouts and maternal family history of problematic alcohol use. Addict Behav.

[33] Pollock VE, Schneider LS, Gabrielli WF, Goodwin DW (1987). Sex of parent and offspring in the transmission of alcoholism. A meta-analysis J Nerv Ment Dis.

[34] Belliveau JM, Stoppard JM (1995). Parental alcohol abuse and gender as predictors of psychopathology in adult children of alcoholics. Addict Behav.

[35] Kendler KS, Gardner CO, Edwards A, Hickman M, Heron J, Macleod J (2013). Dimensions of parental alcohol use/problems and offspring temperament, externalizing behaviors, and alcohol use/problems. Alcohol Clin Exp Res.

[36] Saunders GR, McGue M, Iacono WG, Elkins IJ (2017). Parent-offspring resemblance for drinking behaviors in a longitudinal twin sample. J Stud Alcohol Drugs.

[37] Swedish National Board of Health and Welfare. Dropout in and quality of the medical birth register. 2018. https://www.socialstyrelsen.se/statistik-och-data/register/alla-register/medicinska-fodelseregistret/bortfall-och-kvalitet/. Accessed 2 July 2020.

[38] Statistics Sweden. Multi-generation register. n.d. https://www.scb.se/vara-tjanster/bestalla-mikrodata/vilka-mikrodata-finns/individregister/flergenerationsregistret/. Accessed 26 June 2020.

[39] Ludvigsson JF, Andersson E, Ekbom A, Feychting M, Kim JL, Reuterwall C (2011). External review and validation of the Swedish national inpatient register. BMC Public Health.

[40] Bender R, Lange S (2001). Adjusting for multiple testing–when and how?. J Clin Epidemiol.

[41] Kelly JF, Humphreys K, Ferri M (2020). Alcoholics Anonymous and other 12-step programs for alcohol use disorder. Cochrane Database Syst Rev.

[42] Alonso J, Angermeyer MC, Bernert S, Bruffaerts R, Brugha TS, Bryson H (2004). Use of mental health services in europe: results from the european study of the epidemiology of mental disorders (ESEMeD) project. Acta Psychiatr Scand Suppl.

[43] Cohen E, Feinn R, Arias A, Kranzler HR (2007). Alcohol treatment utilization: findings from the national epidemiologic survey on alcohol and related conditions. Drug Alcohol Depend.

[44] Rehm J, Shield K, Rehm MX, Gmel G, Frick U. Alcohol consumption, alcohol dependence and attributable burden of disease in Europe: Potential gains from effective interventions for alcohol dependence. Toronto, Canada: Centre for Addiction and Mental Health; 2012.

[45] Hjern A, Manhica H (2013). Children as dependents of patients in health care: how many are there?.

[46] Haugland SH, Coombes L, Strandheim A (2020). Parental alcohol intoxication and adverse health outcomes among offspring. A 4-year follow up HUNT study among 2399 Norwegian adolescents. Prev Med Rep..

[47] Manning V, Best DW, Faulkner N, Titherington E (2009). New estimates of the number of children living with substance misusing parents: results from UK national household surveys. BMC Public Health.

[48] Walters RK, Polimanti R, Johnson EC, McClintick JN, Adams MJ, Adkins AE (2018). Transancestral GWAS of alcohol dependence reveals common genetic underpinnings with psychiatric disorders. Nat Neurosci.

[49] Meier SM, Deckert J (2019). Genetics of anxiety disorders. Curr Psychiatry Rep.

[50] Tielbeek JJ, Johansson A, Polderman TJC, Rautiainen MR, Jansen P, Taylor M (2017). Genome-wide association studies of a broad spectrum of antisocial behavior. JAMA Psychiat.

[51] Meier SM, Mattheisen M, Mors O, Mortensen PB, Laursen TM, Penninx BW (2016). Increased mortality among people with anxiety disorders: total population study. Br J Psychiatry.

[52] Krasnova A, Eaton WW, Samuels JF (2019). Antisocial personality and risks of cause-specific mortality: results from the epidemiologic catchment area study with 27 years of follow-up. Soc Psychiatry Psychiatr Epidemiol.

[53] Collins SE (2016). Associations between socioeconomic factors and alcohol outcomes. Alcohol Res.

[54] French MT, Maclean JC, Sindelar JL, Fang H (2011). The morning after: alcohol misuse and employment problems. Appl Econ.

[55] Goldstein RB, Dawson DA, Chou SP, Grant BF (2012). Sex differences in prevalence and comorbidity of alcohol and drug use disorders: results from wave 2 of the national epidemiologic survey on alcohol and related conditions. J Stud Alcohol Drugs.

[56] Lindhagen Å, Linde A. Gender equality policy in Sweden. Government Offices of Sweden.

[57] Holst C, Tolstrup JS, Sørensen HJ, Becker U (2020). Family structure and alcohol use disorder: a register-based cohort study among offspring with and without parental alcohol use disorder. Addiction.

[58] Rehm J (2011). The risks associated with alcohol use and alcoholism. Alcohol Res Health.

[59] National Institute for Health and Care Excellence. Dementia, disability and frailty in later life – mid-life approaches to delay or prevent onset. United Kingdom 2015.

[60] Zilkens RR, Bruce DG, Duke J, Spilsbury K, Semmens JB (2014). Severe psychiatric disorders in mid-life and risk of dementia in late- life (age 65–84 years): a population based case-control study. Curr Alzheimer Res.

