# Supplementary material for: Mortality in adult children of parents with alcohol use disorder: a nationwide register study
Source: Eur J Epidemiol. 2022 Jun 23;37(8):815–26. doi: 10.1007/s10654-022-00883-4 (PMC9463262; doi:10.1007/s10654-022-00883-4)
Supplement: Supplementary file 1 — Supplementary file1 (DOCX 25 KB) [file 10654_2022_883_MOESM1_ESM.docx]

| **Additional file 1.** Swedish ICD codes used to define parental alcohol use disorder and their international equivalents | |
| --- | --- |
| *Swedish ICD codes* | *International equivalents* |
| **ICD-10 code F10**[1] | **ICD-10 code F10**[1] |
| F10.0 acute intoxication | F10.0 acute intoxication |
| F10.1 harmful use | F10.1 harmful use |
| F10.2 dependence syndrome | F10.2 dependence syndrome |
| F10.3 withdrawal state | F10.3 withdrawal state |
| F10.4 withdrawal state with delirium | F10.4 withdrawal state with delirium |
| F10.5 psychotic disorder | F10.5 psychotic disorder |
| F10.6 amnesic syndrome | F10.6 amnesic syndrome |
| F10.7 residual and late onset psychotic disorder | F10.7 residual and late onset psychotic disorder |
| F10.8 other mental and behavioral disorders | F10.8 other mental and behavioral disorders |
| F10.9 unspecified mental and behavioral disorders | F10.9 unspecified mental and behavioral disorders |
|  |  |
| **ICD-9**[2, 3] | **ICD-9**[4] |
| 291A Delirium tremens | 291.0 Delirium tremens |
| 291B Korsakovs psykos, alkoholbetingad | 291.1 Korsakov’s psychosis, alcoholic |
| 291C Alkoholbetingad demens annan än 291A-B | 291.2 Other alcoholic dementia |
| 291D Alkoholhallucinos | 291.3 Other alcoholic hallucinosis |
| 291E Patologiskt alkoholrus | 291.4 Pathological drunkenness |
| 291F Patologisk svartsjuka (Alkoholbetingad paranoia) | 291.5 Alcoholic jealousy |
| 291W Specificerad alkoholpsykos annan än 291A-F | 291.8 Other alcoholic psychosis |
| 291X Alkoholpsykoser, ospecificerade | 291.9 Alcoholic psychosis, unspecified |
| 305A Alkoholmissbruk | 305.0 Nondependent abuse of alcohol |
| 303X Alkoholberoende | 303 Alcohol dependence syndrome |
|  |  |
| **ICD-8**[5] | **ICD-8**[4] |
| 291,00 Delirium tremens | 291.0 Delirium tremens |
| 291,10 Psychosis korsakow (alcoholica) | 291.1 Korsakov’s psychosis (alcoholic) |
| 291,20 Hallucinosis alcoholica alia | 291.2 Other alcoholic hallucinosis |
| 291,30 Paranoia alcoholica | 291.3 Alcoholic paranoia |
| 291,98 Psychosis alcoholica alia definita | 291.9 Other and unspecified alcoholic psychoses |
| 291,99 Psychosis alcoholica nud |  |
| 303,00 Alcoholismus episodicus | 303.0 Episodic excessive drinking |
| 303,10 Alcoholismus habitualis | 303.1 Habitual excessive drinking |
| 303,20 Alcoholismus chronicus ("alcoholic addiction") (dipsomania) | 302.2 Alcohol addiction |
| 303,98 Alcoholismus alius definitus | 303.9 Other and unspecified alcoholism |
| 303,99 Alcoholismus nud |  |

**References**

1. World Health Organization. International Statistical Classification of Diseases and Related Health Problems 10th Revision (Browser). 2019. <https://icd.who.int/browse10/2019/en>. Accessed May 17 2021.

2. Swedish National Board of Health and Welfare. ICD-9 Classification of diseases 1987 (KS87) (1987-1996) codes and code text in Excel format. In: Historic classifications (ICD). Swedish National Board of Health and Welfare. 2019. <https://www.socialstyrelsen.se/utveckla-verksamhet/e-halsa/klassificering-och-koder/icd-10/historiska-klassifikationer/>. Accessed May 10 2021.

3. Swedish National Board of Health and Welfare. Classification of Diseases 1987: Systematic List - Swedish version of the International Classification of Disease, Ninth Revision (ICD-9). Stockholm Liber; 1986.

4. van Drimmelen J. The ICD-10 Classification of Mental and Behavioural Disorders: Conversion Tables between ICD-8, ICD-9 and ICD-10. Geneva: Division of Mental Health of the World Health Organization 1994.

5. Swedish National Board of Health and Welfare. ICD-8 Classification of diseases etc. 1968 (1969-1986) codes and code text in Excel format. In: Historical classifications. Swedish National Board of Health and Welfare. 2019. <https://www.socialstyrelsen.se/utveckla-verksamhet/e-halsa/klassificering-och-koder/icd-10/historiska-klassifikationer/>. Accessed May 23, 2021 2021.
