# Supplementary material for: Mortality in adult children of parents with alcohol use disorder: a nationwide register study
Source: Eur J Epidemiol. 2022 Jun 23;37(8):815–26. doi: 10.1007/s10654-022-00883-4 (PMC9463262; doi:10.1007/s10654-022-00883-4)
Supplement: Supplementary file 2 — Supplementary file2 (DOCX 28 KB) [file 10654_2022_883_MOESM2_ESM.docx]

| **Additional file 2.** ICD-8, ICD-9, and ICD-10 codes for alcohol- and drug-related deaths^a^ | | | | | |
| --- | --- | --- | --- | --- | --- |
| ***Definition*** | ***ICD-10*** | ***ICD-9*** |  | ***Definition*** | ***ICD-8*** |
| **Alcohol-related causes of death** |  |  |  | **Alcohol-related causes of death** |  |
| Alcohol-induced pseudo-Cushing syndrome | E24.4 | 2500 |  | Delirium tremens | 291.00 |
| Degeneration of nervous system due to alcohol | G31.2 | 303 |  | Korsakov’s psychosis, alcoholic | 291.10 |
| Alcoholic polyneuropathy | G62.1 | 3575 |  | Other alcoholic hallucinosis | 191.20 |
| Alcoholic myopathy | G72.1 | 3594 |  | Alcoholic paranoia | 291.30 |
| Alcoholic cardiomyopathy | I42.6 | 4255 |  | Other^b^ | 291.98 |
| Mental and behavioral disorders due to use of alcohol | F10 | 291, 2922, 3050 |  | Non ultra descriptus (NUD)/unspecified^b^ | 291.99 |
| Alcoholic gastritis | K29.2 | 3553 |  | Episodic excessive drinking | 303.00 |
| Alcohol-induced chronic pancreatitis | K86.0 | 5771 |  | Habitual excessive drinking | 303.10 |
| Alcoholic liver disease | K70 | 5710, 5711, 5712, 5713, 5728 |  | Alcoholic addiction (Dipsomania, Chronic alcoholism, Chronic ethylism) | 303.20 |
| Maternal care for (suspected) damage to fetus from alcohol | O35.4 | 6554 |  | Other^c^ | 303.98 |
| Fetus and newborn affected by maternal use of alcohol | P04.3 | 7607 |  | Unspecified^c^ | 303.99 |
| Fetal alcohol syndrome (dysmorphic) | Q86.0 | 7598 |  | Cirrhosis of the liver, alcoholic | 571.00 |
| Poisoning by and exposure to alcohol, undetermined intent | Y15 | E9809 |  | Fatty degeneration of the liver, alcoholic^d^ | 571.01 |
| Intentional self-poisoning by and exposure to alcohol | X65 | E9509 |  | *Alcohol in combination with specified medical agents* | |
| **Drug-related causes of death** |  |  |  | Alcohol with opium derivatives | 979.00 |
| Mental and behavioral disorders due to use of opioids | F11 | 3055,3040, 2920, 2921, 2940, 2928 |  | Alcohol with barbiturates | 979.10 |
| Mental and behavioral disorders due to use of cannabinoids | F12 | 2922, 3052, 3043, 2920, 2921, 2940, 2928, 2929 |  | Alcohol with non-barbiturate sedatives | 979.20 |
| Mental and behavioral disorders due to use of sedatives or hypnotics | F13 | 2922, 3054, 3041, 3046, 2920, 2921, 2940, 2928, 2929 |  | Alcohol with antihistamines | 979.30 |
| Mental and behavioral disorders due to use of cocaine | F14 | 2922, 3056, 3042, 2920, 2921, 2940, 2928, 2929 |  | Alcohol with psychopharmacologic agents | 979.40 |
| Mental and behavioral disorders due to use of other stimulants, including caffeine | F15 | 2929, 3057, 3059, 3044, 2920, 2921, 2940, 2928, 2929 |  | *Toxic effect of alcohol* |  |
| Mental and behavioral disorders due to use of hallucinogens | F16 | 2922, 3053, 3045, 2920, 2921, 2940, 2928, 2929 |  | Ethyl alcohol | 980.00 |
| Mental and behavioral disorders due to use of volatile solvents | F18 | 2922, 2928, 3059, 2922, 3046, 2920, 2921, 2940, 2928, 2929 |  | Industrial substances that contain ethyl alcohol (alcohol surrogates)^e^ | 980.01 |
| Mental and behavioral disorders due to multiple drug use and use of other psychoactive substances | F19 | 2922, 2928, 3059, 2922, 2928, 3059, 3046 |  | Methyl alcohol | 980.10 |
| Maternal care for (suspected) damage to fetus by drugs | O35.5 | 6555 |  | Isopropyl alcohol | 980.20 |
| Fetus and newborn affected by maternal use of drugs of addiction | P04.4 | 7607 (X?) |  | Other alcohol^e^ | 980.98 |
| Poisoning by narcotics and psychodysleptics [hallucinogens] | T40 | 9650, 9657, 9658, 9754, 9685, 9779, 9696 |  | Alcohol unspecified^e^ | 980.99 |
| Psychostimulants with abuse potential | T43.6 | 9697 |  | **Drug-related causes of death** |  |
| Other and unspecified drugs, medicaments and biological substances | T50.9 | 9774 |  | *Drug dependence* |  |
| Accidental poisoning by and exposure to antiepileptic, sedative-hypnotic, antiparkinsonism and psychotropic drugs, not elsewhere classified | X41 | E851... |  | Opium, opium alkaloids, and the derivatives | 304.00 |
| Accidental poisoning by and exposure to narcotics and psychodysleptics [hallucinogens], not elsewhere classified | X42 | E8500… |  | Synthetic analgesics with morphine-like effects | 304.10 |
| Accidental poisoning by and exposure to other drugs acting on the autonomic nervous system | X43 | E8530… |  | Barbiturates | 304.20 |
| Accidental poisoning by and exposure to other and unspecified drugs, medicaments and biological substances | X44 | E8500… |  | Other hypnotics and sedatives or tranquilizers | 304.30 |
| Intentional self-poisoning by and exposure to nonopioid analgesics, antipyretics and antirheumatics | X60 | E950 |  | Cocaine | 304.40 |
| Intentional self-poisoning by and exposure to antiepileptic, sedative-hypnotic, antiparkinsonism and psychotropic drugs, not elsewhere classified | X61 | E950 |  | Cannabis sativa | 304.50 |
| Intentional self-poisoning by and exposure to narcotics and psychodysleptics [hallucinogens], not elsewhere classified | X62 | E950 |  | Other psycho-stimulants | 304.60 |
| Intentional self-poisoning by and exposure to other drugs acting on the autonomic nervous system | X63 | E950 |  | Hallucinogenics | 304.70 |
| Intentional self-poisoning by and exposure to other and unspecified drugs, medicaments and biological substances | X64 | E950 |  | Other | 304.88 |
| Poisoning by and exposure to nonopioid analgesics, antipyretics and antirheumatics, undetermined intent | Y10 | E9800 |  | Unspecified | 304.99 |
| Poisoning by and exposure to antiepileptic, sedative-hypnotic, antiparkinsonism and psychotropic drugs, not elsewhere classified, undetermined intent | Y11 | E9801, E9802, E9803, E9804, |  |  |  |
| Poisoning by and exposure to narcotics and psychodysleptics [hallucinogens], not elsewhere classified, undetermined intent | Y12 | E9800, E9803, E9804 |  |  |  |
| Poisoning by and exposure to other drugs acting on the autonomic nervous system, undetermined intent | Y13 | E9803, E9804 |  |  |  |
| Poisoning by and exposure to other and unspecified drugs, medicaments and biological substances, undetermined intent | Y14 | E9800, E9801, E9802, E9803, E9804, E9805, E9806, E9809 |  |  |  |
| ^a^Swedish codes in this list obtained from the website of the Swedish National Board of Health and Welfare [1].  ^b^This code would fall under code 291.9, Other and unspecified in the Eighth Revision International Classification of Diseases, Adapted for Use in the United States, which covered “Alcoholic: dementia, insanity, mania; Alcoholism (chronic) with psychosis: Psychosis, alcoholic”[2].  ^c^This code would fall under code 303.9, Other and unspecified alcoholism in the Eighth Revision International Classification of Diseases, Adapted for Use in the United States [2].  ^d^This code would fall under code 571.0, Cirrhosis of the liver, alcoholic in in the Eighth Revision International Classification of Diseases, Adapted for Use in the United States [2].  ^e^This code would fall under code 980.9, Other (alcohol), in the Eighth Revision International Classification of Diseases, Adapted for Use in the United States, which covered “NOS, amyl, butyl, denatured, industrial” [2]. | | | | | |

**References**

1. Swedish National Board of Health and Welfare. Historical classifications (ICD). 2019. www.socialstyrelsen.se.

2. U.S. Department of Health E, and Welfare Public Health Service National Center for Health Statistics. Eighth Revision International Classification of Diseases, Adapted for Use in the United States. vol 1, Tabular List. Washington, D.C.: U.S. Government Printing Office; ca. 1965.
