# Supplementary material for: Mortality in adult children of parents with alcohol use disorder: a nationwide register study
Source: Eur J Epidemiol. 2022 Jun 23;37(8):815–26. doi: 10.1007/s10654-022-00883-4 (PMC9463262; doi:10.1007/s10654-022-00883-4)
Supplement: Supplementary file 3 — Supplementary file3 (DOCX 23 KB) [file 10654_2022_883_MOESM3_ESM.docx]

| **Additional file 3.** Adjusted hazard ratios and 95% confidence intervals of death in firstborns^a^ in the study population by cause and parental alcohol use disorder | | | | | | |
| --- | --- | --- | --- | --- | --- | --- |
|  | | | *Neither parent had AUD* | *≥1 parent had AUD* | *Mother had AUD* | *Father had AUD* |
| *Number* | | | 1,321,273 | 78,726^b^ | 28,106 | 55,632 |
| *Cause of death* | | |  |  |  |  |
|  | *Any cause* | | 1.0 | 1.38 (1.32-1.44) | 1.65 (1.55-1.76) | 1.24 (1.18-1.31) |
|  | *Medical causes* | | 1.0 | 1.03 (0.97-1.10) | 1.29 (1.18-1.41) | 0.91 (0.85-0.98) |
|  | *External causes of*  *injuries and poisoning* | | 1.0 | 2.05 (1.93-2.18) | 2.31 (2.11-2.53) | 1.87 (1.74-2.00) |
|  |  | *Suicide* | 1.0 | 2.20 (1.97-2.45) | 2.61 (2.24-3.05) | 1.91 (1.68-2.17) |
|  |  | *Assault* | 1.0 | 1.80 (1.36-2.39) | 1.99 (1.31-3.02) | 1.64 (1.18-2.27) |
|  |  | *Accident* | 1.0 | 2.00 (1.85-2.16) | 2.19 (1.95-2.45) | 1.86 (1.70-2.03) |
|  | *Alcohol-related causes* | | 1.0 | 1.35 (0.75-2.43) | 1.56 (0.63-3.83) | 1.55 (0.83-2.92) |
|  | *Drug-related causes* | | 1.0 | 3.09 (2.80-3.40) | 3.27 (2.84-3.76) | 2.79 (2.50-3.12) |
|  | *Drug-related causes excluding accidential poisonings*^c^ | | 1.0 | 3.15 (2.75-3.61) | 3.52 (2.91-4.25) | 2.76 (2.36-3.22) |
| Adjusted for year of birth, sex, highest level of parental education, and loss of a parent in childhood.  ^a^A firstborn was defined as the first child born to each mother-father pair. Firstborns constituted 1,399,999 of the 2,421,479 live-born children born in Sweden between January 1, 1973, and December 31, 1995.  ^b^For 5012 firstborns, both parents had AUD.  ^c^Accidental poisonings: ICD-8: 304.00-304.30; ICD-9: E8500, E851, E8530; ICD-10: X41-X44. | | | | | | |
